# Late-onset hypercyanotic spells in duct-dominant Tetralogy of Fallot with pulmonary atresia from juxtaductal PDA–LPA stenosis: CT angiography for preoperative mapping

**DOI:** 10.1093/jscr/rjag274

**Published:** 2026-04-17

**Authors:** Aditya Pachwa, Siddartha R Gangapuram, Pranav R Pariyada, Lakshya N Samineni

**Affiliations:** Mamata Academy of Medical Sciences, Hyderabad 500118, India; Mamata Academy of Medical Sciences, Hyderabad 500118, India; Mamata Academy of Medical Sciences, Hyderabad 500118, India; College of Medical Sciences Teaching Hospital, Chitwan, Bharatpur 44200, Nepal

**Keywords:** Tetralogy of Fallot, pulmonary atresia with ventricular septal defect, patent ductus arteriosus, pulmonary artery stenosis, computed tomography angiography, preoperative mapping

## Abstract

Tetralogy of Fallot with pulmonary atresia (ToF-PA) requires precise delineation of extracardiac pulmonary blood supply to guide optimal palliation, especially in duct-dependent physiology. We report an 8-month-old infant with late-onset central cyanosis and recurrent cry-triggered hyper cyanotic spells. Computed tomography (CT) thoracic angiography showed classic ToF with short-segment pulmonary atresia, a malaligned perimembranous ventricular septal defect, and hypoplastic yet confluent branch pulmonary arteries. A long, tortuous patent ductus arteriosus (PDA) provided dominant pulmonary flow, with severe focal juxtaductal stenosis just before insertion into the left pulmonary artery and reduced distal pulmonary arborization. A small ostium secundum atrial septal defect was identified. Minor systemic collaterals were seen, without large dominant major aortopulmonary collateral arteries, suggesting duct-dominant physiology potentially amenable to pulmonary artery rehabilitation. This case highlights late deterioration from progressive PDA–left pulmonary artery narrowing and underscores CT angiography as a key decision map for catheter or surgical palliation and operative planning.

## Introduction

Tetralogy of Fallot with pulmonary atresia (ToF-PA) is a severe cyanotic congenital heart disease with absent antegrade right ventricular outflow to the pulmonary arteries. Pulmonary blood flow therefore, depends on a patent ductus arteriosus (PDA) and/or systemic-to-pulmonary collateral vessels [1]. Because anatomy varies widely, management is dictated by pulmonary artery confluence and size, distal arborization, collateral supply pattern, and ductal morphology and insertion [2]. Preoperative anatomic mapping of the duct, branch pulmonary arteries, and collateral burden is essential for planning palliation and later repair [3]. Computed tomography angiography (CTA) provides comprehensive preoperative evaluation when echocardiography is limited, particularly for ductal tortuosity, juxtaductal stenosis, distal pulmonary arteries, and collateral anatomy [4]. We report an infant with late-onset cyanosis and cry-triggered spells due to duct-dominant ToF-PA with a tortuous stenotic PDA inserting into hypoplastic but confluent pulmonary arteries, small systemic collaterals, and a small ostium secundum atrial septal defect (ASD).

## Case presentation

**Figure 1:**
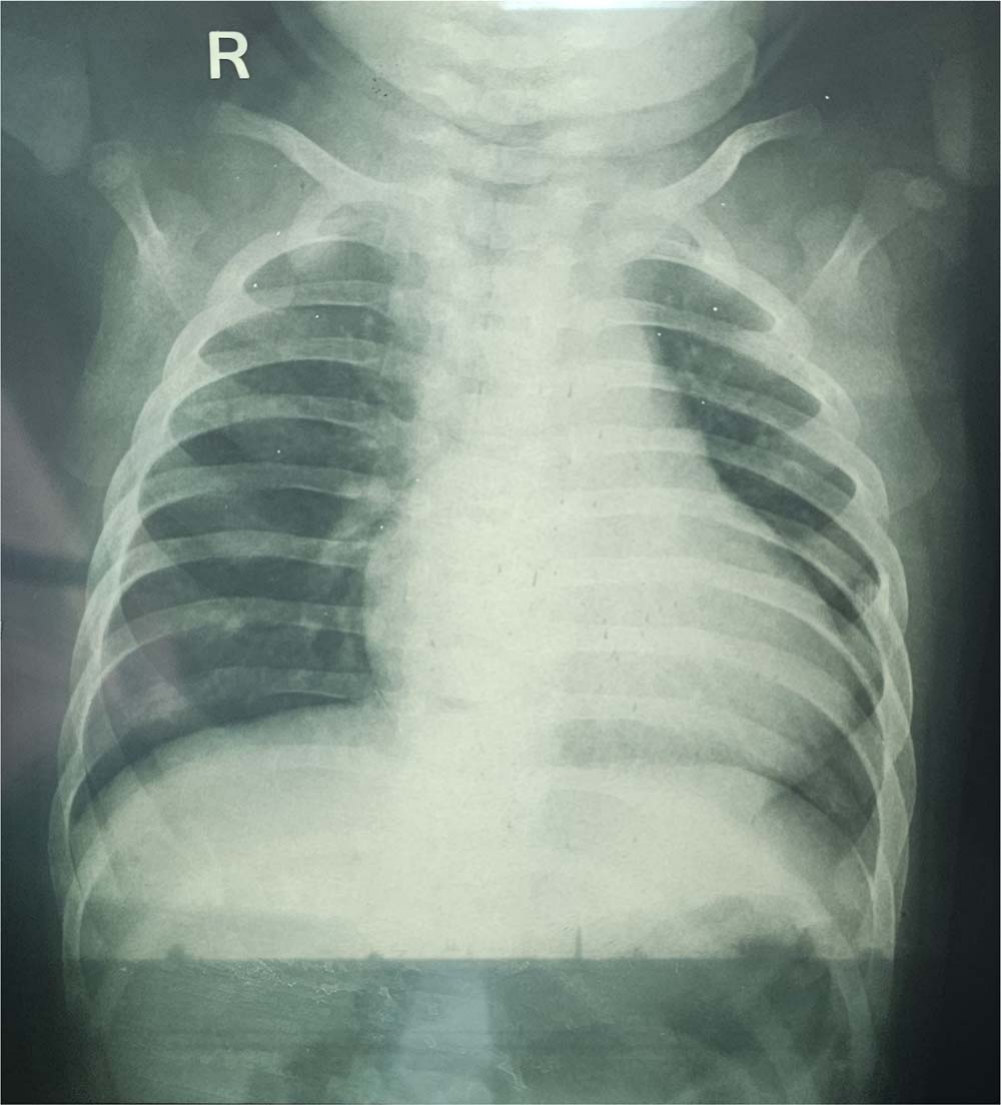
Chest radiograph shows mild cardiomegaly with decreased pulmonary vascular markings and clear lung fields.

An 8-month-old infant presented with recurrent cyanotic spells precipitated by crying for 1 month; caregivers denied prior recognizable signs. There was no fever, respiratory distress, trauma, hospitalizations, or any prostaglandin E1 exposure. On examination, infant weighed 7 kgs and length 60 cm. The infant was active but found to have central cyanosis and pallor; heart rate was 138/min and room-air SpO₂ was 78%. The precordium was quiet without heaves or thrills; heart sounds revealed an ejection systolic murmur. There was no hepatomegaly. Laboratory evaluation showed hemoglobin 9.2 g/dL and leucocytosis (18.3 × 10^3^/μL). Chest radiograph showed mild cardiomegaly with decreased pulmonary vascular markings and clear lung fields (Fig. 1). Electrocardiogram demonstrated sinus tachycardia with right-axis deviation and right ventricular hypertrophy.

**Figure 2:**
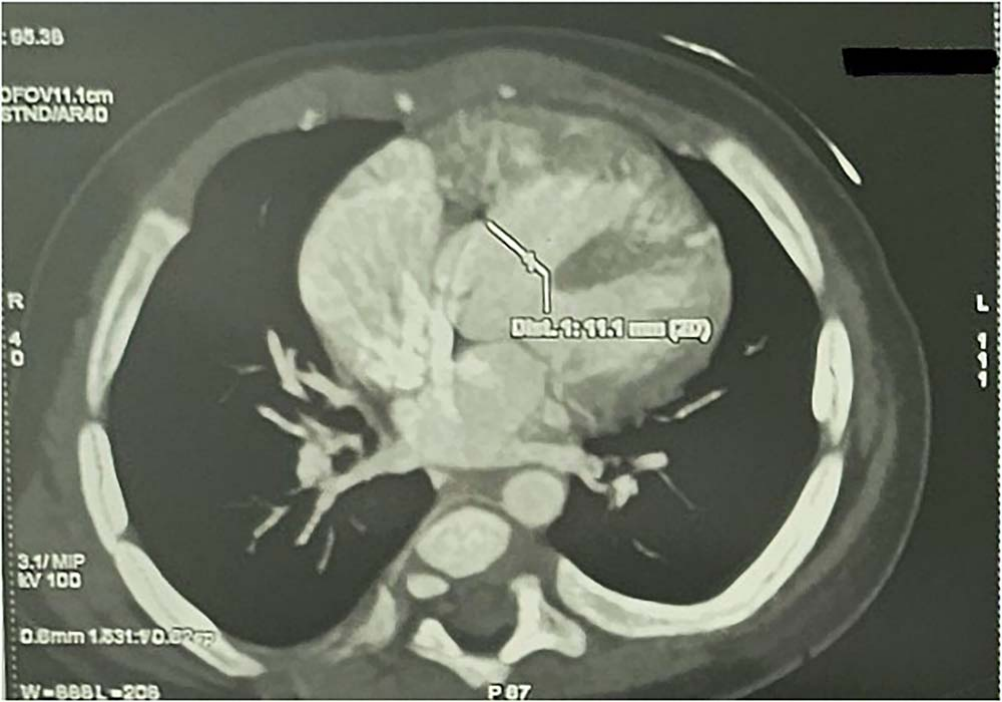
CTA demonstrating malaligned perimembranous ventricular septal defect with ToF morphology.

**Figure 3:**
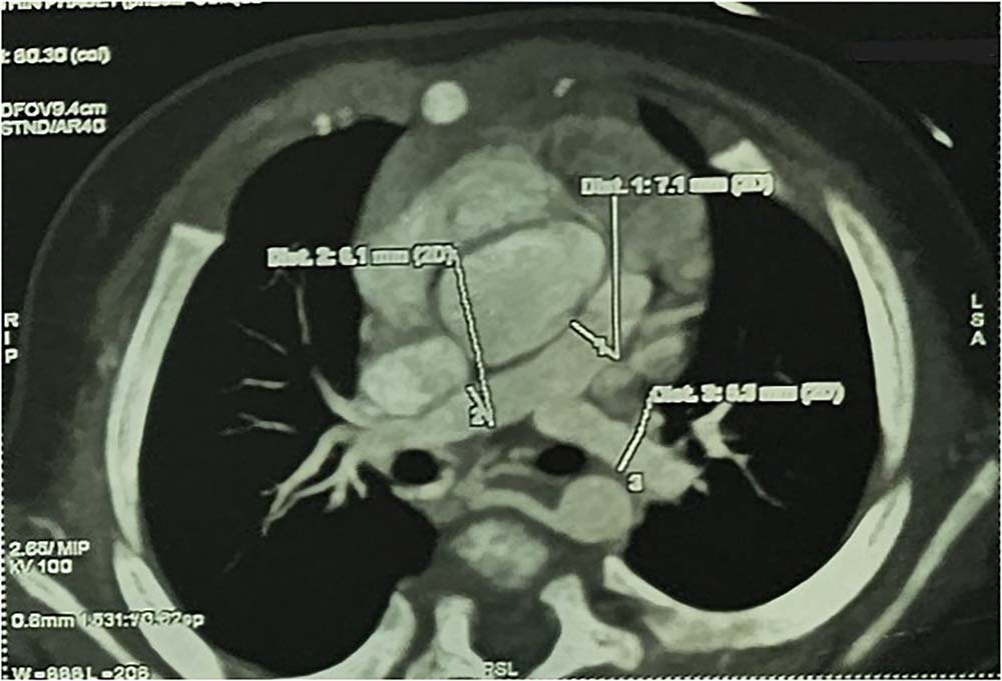
CTA demonstrating hypoplastic MPA with confluent hypoplastic branch PAs.

**Figure 4:**
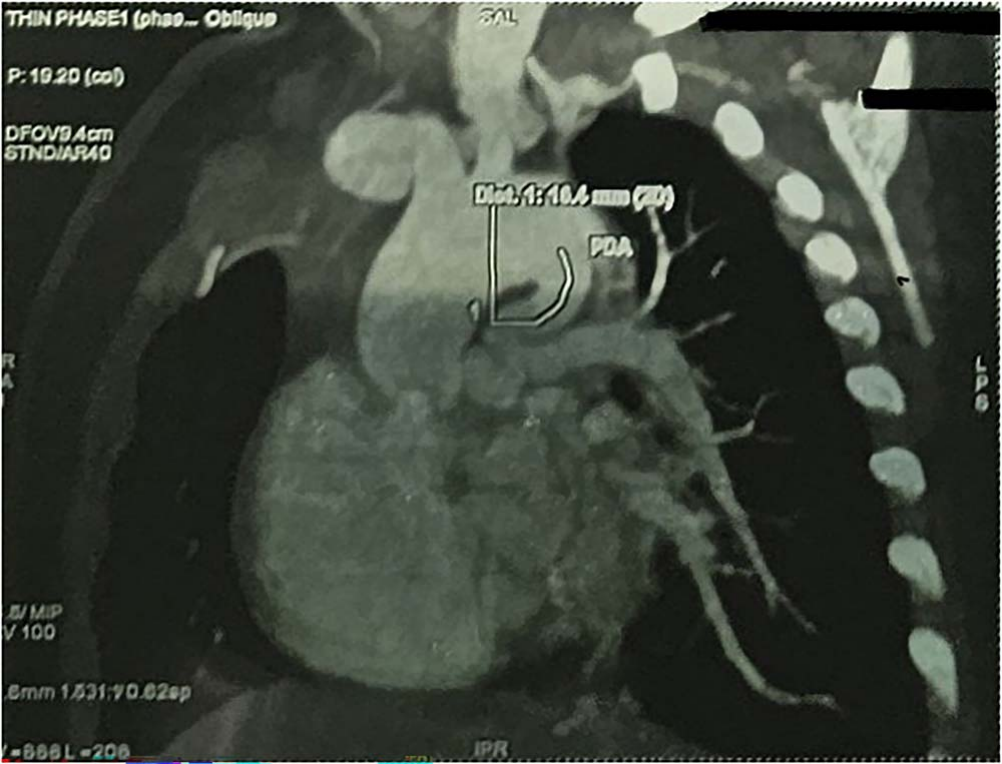
CTA demonstrating long tortuous PDA supplying the pulmonary circulation.

**Figure 5:**
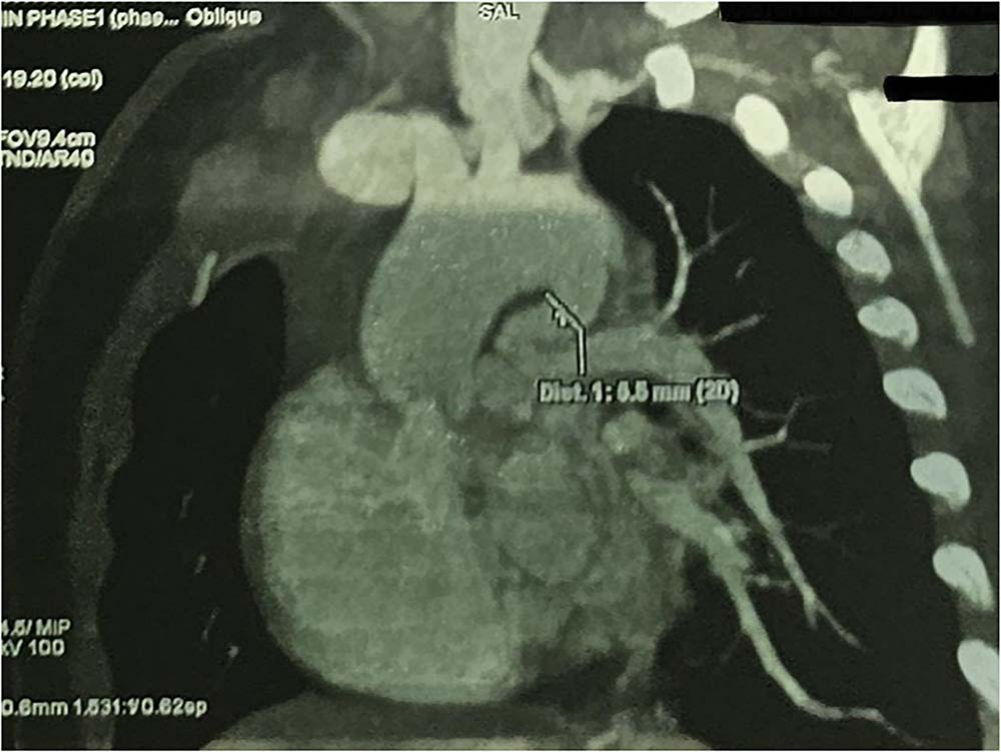
CTA demonstrating max PDA caliber with focal narrowing near LPA insertion.

**Figure 6:**
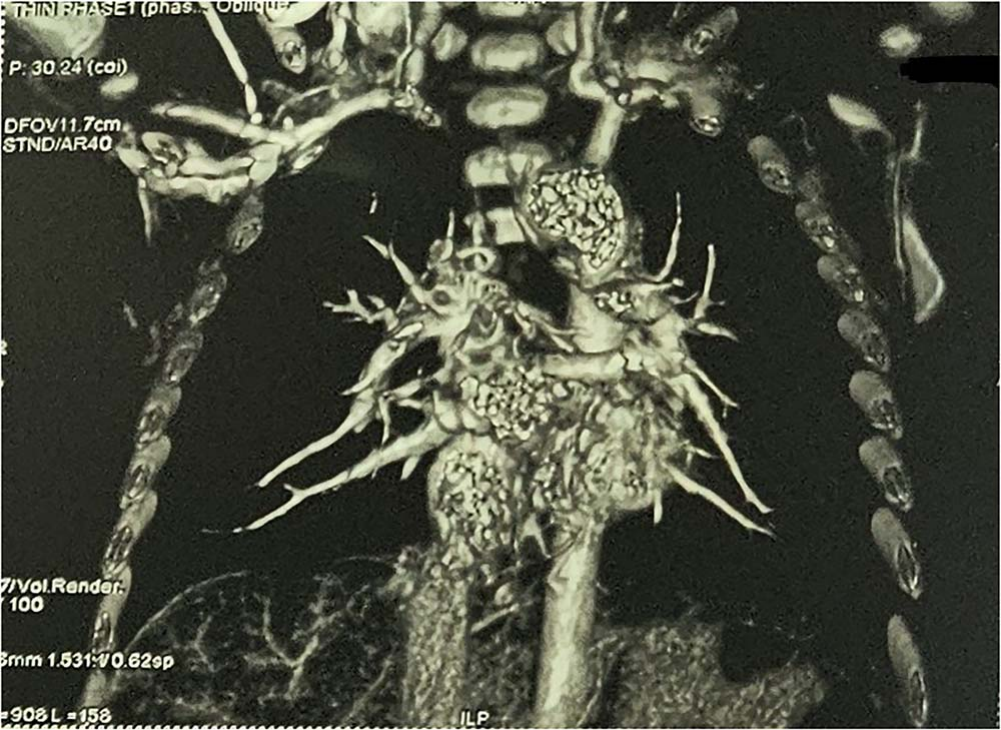
CTA demonstrating reduced distal pulmonary arborization.

CTA demonstrated situs solitus, D-loop, levocardia, and side-by-side great arteries with normal coronary origins. Intracardiac anatomy showed ToF morphology with short-segment pulmonary atresia and a malaligned perimembranous ventricular septal defect (11.1 mm) with <50% aortic override, with moderate right-sided chamber enlargement and right ventricular hypertrophy (Fig. 2). The main pulmonary artery was hypoplastic (7.1 mm) with confluent hypoplastic branch pulmonary arteries (right pulmonary artery 6.1 mm; left pulmonary artery (LPA) 5.3 mm) (Fig. 3). A long, tortuous PDA supplied the pulmonary circulation (Fig. 4). The duct measured 16.4 mm in length with a maximal diameter of 5.5 mm and severe focal stenosis immediately prior to insertion into the left pulmonary artery; distal pulmonary arborization was reduced bilaterally (Figs 5 and 6). A small ostium secundum ASD was present. Multiple mildly dilated tortuous intercostobronchial collaterals were seen in the mediastinum; the largest arose at the D4–D5 level on the right (2.9 mm) supplying right lung parenchyma. An additional 2 mm systemic collateral from the celiac trunk supplied bilateral lower-lobe parenchyma. No large dominant major aortopulmonary collateral arteries (MAPCAs) were identified. Systemic and pulmonary venous returns were normal.

Hospital management was supportive. Spells were treated with knee–chest positioning, calming, and supplemental oxygen, with stabilization and no further in-hospital events. In view of leucocytosis, oral amoxicillin–clavulanate was started while evaluating for intercurrent infection. Paracetamol was given as needed. Oral iron and multivitamins were initiated for anemia. After multidisciplinary discussion, staged palliation was chosen, and PDA stenting was performed with relief of juxtaductal LPA stenosis and improvement in resting oxygen saturation, with resolution of spells.

## Discussion

CTA was decisive because the pivotal management questions were extracardiac and geometric: is pulmonary blood flow duct-dominant versus MAPCA-dominant (favoring pulmonary artery rehabilitation versus unifocalization), and is the duct suitable for stenting based on origin, length, tortuosity, focal stenosis, and its relationship to branch pulmonary artery origins? In our patient, CTA showed mildly enlarged intercostobronchial channels and a small celiac-origin collateral without surgically dominant MAPCAs, supporting duct-dominant physiology with confluent central pulmonary arteries potentially recruitable toward later definitive repair [3].

CTA concurrently defines ductal geometry, branch pulmonary artery caliber, distal arborization, and collateral burden, explaining its central role in preoperative mapping for PA-VSD/ToF-PA [5]. The anatomy also aligns with the concept that ductal tissue may extend into the juxtaductal pulmonary artery and contribute to progressive narrowing and branch pulmonary artery compromise (‘pulmonary ductal coarctation’), particularly at the proximal LPA [6]. Clinically, months without obvious cyanosis followed by new spells are consistent with progressive restriction at the PDA–LPA junction. Once that fixed lesion became critical, crying likely increased oxygen demand and transiently altered systemic and pulmonary vascular resistances, further reducing effective flow across a fixed bottleneck and precipitating episodic severe desaturation.

In classic ToF, spells are typically attributed to dynamic right ventricular outflow obstruction causing an acute fall in pulmonary blood flow, amplification of right-to-left shunting, and impaired systemic oxygen delivery during agitation, crying, dehydration, or anemia [7]. In duct-dependent ToF-PA, however, the duct–branch pulmonary artery junction becomes the rate-limiting lesion to identify and treat; CTA provides the anatomic roadmap for that decision and for anticipating branch pulmonary artery compromise. PDA stenting outcomes correlate strongly with ductal morphology, particularly tortuosity and the propensity for branch pulmonary artery ‘jailing’ at the duct–pulmonary artery junction. In our case, the long tortuous duct with severe pre-insertion stenosis into the LPA implied higher technical risk: challenging wire passage, stable positioning across a tight segment, and possible compromise of adjacent branch flow if protrusion or malapposition occurred near the bifurcation [8]. When anatomy is favorable, stenting can promote growth of hypoplastic but confluent pulmonary arteries targeted for rehabilitation [9].

For a duct like ours—tortuous with severe juxtaductal narrowing—CTA therefore functions as the decision map, guiding whether stenting risks outweigh surgical shunting and informing planning for possible branch pulmonary artery plasty at the ductal insertion 6. Two additional points are clinically relevant: First, hemoglobin 9.2 g/dL reduces arterial oxygen content and may lower the physiologic threshold for spells even if SpO₂ appears similar. Second, the small ostium secundum ASD is unlikely to be the primary driver of cyanosis but may modulate desaturation during transient rises in right-sided pressures and should be documented for operative planning.

## Conclusion

Duct-dominant ToF-PA can present beyond early infancy with new-onset cyanosis and cry-triggered hyper cyanotic spells when progressive juxtaductal PDA stenosis critically limits pulmonary blood flow. CTA is pivotal to define ductal morphology and the duct–branch pulmonary artery junction, quantify pulmonary artery confluence and caliber, assess distal arborization, and characterize collateral burden, thereby guiding selection of palliation and operative strategy.
